# Can social media reliably estimate unemployment?

**DOI:** 10.1093/pnasnexus/pgaf309

**Published:** 2025-12-30

**Authors:** Do Lee, Manuel Tonneau, Boris Sobol, Nir Grinberg, Samuel P Fraiberger

**Affiliations:** Department of Economics, New York University, New York, NY 10012, USA; Oxford Internet Institute, University of Oxford, Oxford OX1 3JS, United Kingdom; Development Impact Group, The World Bank, Washington, DC 20433, USA; Department of Computer Science, New York University, New York, NY 10012, USA; Faculty of Computer and Information Science, Ben-Gurion University of the Negev, Beersheba 8410501, Israel; Faculty of Computer and Information Science, Ben-Gurion University of the Negev, Beersheba 8410501, Israel; Development Impact Group, The World Bank, Washington, DC 20433, USA; Department of Computer Science, New York University, New York, NY 10012, USA

**Keywords:** unemployment, social media, natural language processing

## Abstract

Digital trace data hold tremendous potential for measuring policy-relevant outcomes in real-time, yet its reliability is often questioned. Here, we propose a principled yet simple approach: capturing individual disclosures of unemployment using a fine-tuned AI model and post-stratification adjustment using inferred user demographics. We show that our methodology consistently outperforms the industry’s forecasting average and can improve the predictions of US unemployment insurance claims, up to 2 weeks in advance, at the national, state, and city levels at both turbulent and stable times. The results demonstrate the potential of combining AI models with statistical modeling to complement traditional survey methodology, and contribute to better-informed policymaking, especially at turbulent times.

Significance Statement Accurate and timely labor market data are essential for effective policymaking, yet traditional methods often suffer from delays and limited geographic resolution. This study presents an approach to forecasting unemployment insurance claims in the United States by leveraging social media data. Using a fine-tuned AI model to detect self-disclosures of unemployment on social media, combined with statistical adjustments for demographic biases, our methodology delivers real-time insights that outperform traditional industry forecasts. This approach demonstrates the potential of integrating AI models with statistical techniques to complement traditional economic data sources, particularly during periods of economic turbulence.

## Introduction

Two weeks after COVID-19 was officially declared a pandemic, the number of people filing new claims for unemployment benefits (“UI claims”) in the United States surged from about 278 thousand to nearly 6 million. Absent accurate, real-time information about the magnitude of the shock that triggered the worst job crisis since the Great Depression, government agencies across the country quickly became unable to process claims in a timely manner, which had serious economic and psychological ramifications for beneficiaries (1).

This episode epitomizes that timely and disaggregated information about the labor market is vital for economic well-being. It improves market efficiency (2) and enables the design of evidence-based policies (3). However, official statistics are typically available with a considerable lag—especially at high resolution—and are subject to ex post revisions, which impedes policymakers’ ability to alleviate the impact of economic shocks in a timely fashion. For example, US statistics on UI claims are published with a lag of at least 4 days at the national and state levels as well as for a limited number of cities; other valuable unemployment statistics are only available on a monthly or quarterly basis, with limited coverage at the subnational level. These limitations are even more severe in low- and middle-income countries, where national statistical agencies often lack the resources to consistently collect timely and reliable labor market data (4, 5).

In this context, the potential of real-time digital trace data to complement official statistics has been explored extensively over the past decade (6–8). Social media data have proven to be a valuable source of information across various domains such as quantifying migration flows (9), the impact of natural disasters (10), economic mobility and connectedness (11, 12), asset markets fluctuations (13, 14), economic policy uncertainty (15), inflation expectations (16), and employment shocks (17). Several studies have also identified signals from social media—including users’ diurnal rhythm (18), connectedness and keyword counts (19, 20)—that can be correlated with unemployment statistics; however, these approaches fell short of demonstrating sufficient predictive power and robustness to be relied upon in practice.

In this study, we present a principled yet simple methodology for forecasting the number of UI claims in the United States at the national, state, and city levels up to 2 weeks ahead of the official release using unemployment self-disclosures on Twitter. We focus on UI claims as it is the most frequently updated official statistics about the labor market and an important macroeconomic measure for policymakers (21, 22), macroeconomic forecasters (23, 24), and financial markets (25). We identify unemployment disclosures by training a fine-tuned transformer-based classifier using Active Learning (26), a sampling strategy that maximizes detection performance by letting the model choose the data points from which it learns. Our AI model, specifically a Transformer encoder-based classifier from the BERT family, captures substantially more self-disclosures of unemployment on Twitter compared to previous approaches without compromising on precision, it identifies disclosures from more people, and yields a more representative sample of unemployed users. Then, using inferred user demographics and census population estimates, we construct a Twitter unemployment index by post-stratifying the proportion of unemployed users to correct for the sample nonrepresentativeness. The post-stratification adjusts for the fact that Twitter users do not perfectly represent the general population. UI claim predictions are based on an autoregressive model using the Twitter unemployment index, past official statistics, and the industry’s consensus forecast (27) (see Methods). To thoroughly evaluate our methodology, we test model predictions over the course of 3 years, during both turbulent and “normal” times, evaluate accuracy up to 2 weeks before the official statistics are released, measure robustness at the national, state, and city levels, and compare performance to the industry’s leading consensus forecast. By contrasting our models with previously proposed rule-based approaches (19), unweighted variants, and down-sampled versions, we gain insight into the contributing factors for the model’s success. Finally, we demonstrate the model’s ability to fill in gaps in official statistics.

We present this study as a proof of concept for using AI models and social media data to extract timely, granular signals of economic activity. Rather than proposing a ready-to-deploy tool for all contexts and periods, our aim is to offer a flexible methodological framework that can adapt as platforms, user behavior, and data access evolve. The findings highlight the public value of this approach and the potential for partnerships with platforms, where controlled access to anonymized signals could support real-time monitoring while protecting privacy. More broadly, the work underscores the need for responsible data access, whether through regulation or partnerships, to enable research that serves the public interest.

## Methods

Self-disclosures of unemployment status are extremely rare in the sea of social media content. Therefore, we need a large sample of users and a comprehensive approach to detect unemployment self-disclosures. To that end, we query the Twitter API to collect the tweets posted by users with a profile location that uniquely maps to a geographical location in the United States, and snowball sample additional users mentioned in these tweets (see Methods for more details). The dataset analyzed here consists of public tweets posted by a snowball sample of 31.5 million US-based users collected between January 2020 and December 2022. We use two different approaches to identify public self-disclosures of unemployment status in tweets’ text. The first is a rule-based language model inspired by previous work (19), where a tweet is considered disclosing one’s unemployment status if it contains any of 75 theoretically motivated phrases describing job loss such as “I just lost my job” (see Fig. S2 for a complete list of phrases and their prevalence). The second approach trains an AI language model by following the procedure proposed by Tonneau et al. (28). It involves an Active Learning iterative process where at each step, a Transformer encoder-based classifier from the BERT model family (29) is trained on the currently available, manually labeled tweets, and then model uncertainty is used to select additional tweets to be sent for labeling. The final model, referred to hereafter as JoblessBERT and available publicly,^a^ is trained on a set of 8,838 tweets (see Methods for more details). To construct a daily unemployment index, we calculate the percentage of users who disclosed their employment status (using either the rule-based or JoblessBERT model) out of all active users observed in a sliding window of 7 days. We also construct post-stratified versions of the index to adjust for the platform’s nonrepresentative user base (30) by reweighting users based on inferred age, gender, and location from their profiles to match US general population estimates from the Census Bureau (see Methods and Fig. S1). We use a deep-learning model (31) to infer user age and gender using the profile images and metadata. Users without valid demographic inferences are retained in the analysis, as missing attributes are imputed by sampling from the distribution of users with observed demographics, stratified by state (Methods). Finally, we use an autoregressive distributed lag model to predict weekly UI claims, where covariates consist of the Twitter unemployment index, official statistics about past UI claims, and the industry’s consensus forecast when it becomes available (full model specifications are in the Methods). The autoregressive model is trained on data from a weekly sample of 208 observations spanning 2016–2019 (inclusive) and tested in a weekly sample of 156 observations spanning 2020–2022 (inclusive), which includes the turbulent times of the COVID-19 pandemic. To ensure that the covariates are expressed in comparable units, the UI claims and consensus forecasts are normalized by the size of the labor force during the previous month. Hereafter, we refer to the normalized UI claims as UI claims for brevity.

## Results

### Detecting disclosures of unemployment status

**Fig. 1.**
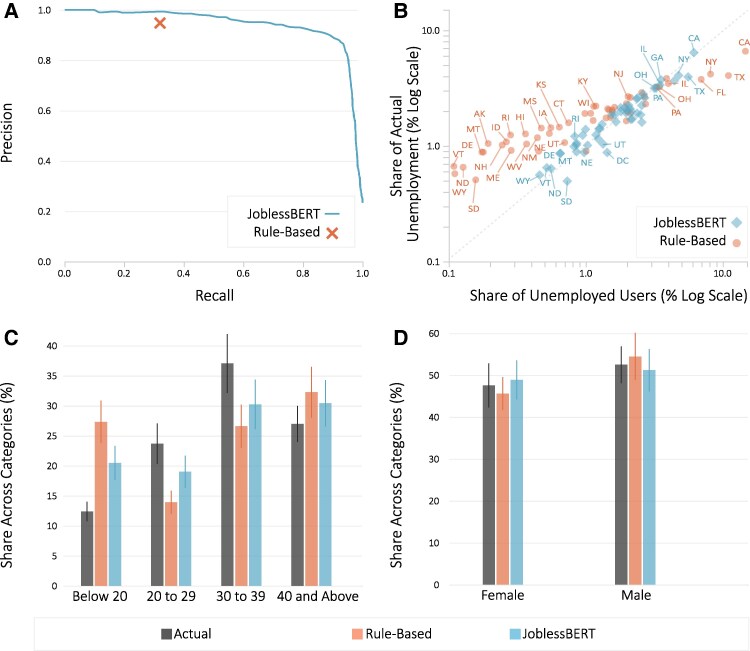
Detecting disclosures of unemployment status. A) Precision-recall curve comparing JoblessBERT and the rule-based model in detecting unemployment self-disclosures. B–D) Comparison of the distribution of unemployed users and actual unemployment rates across US states (B), age brackets (C), and gender (D). Precision-recall curve has been computed using an evaluation sample of 3,546 tweets produced in prior work (28). State-level distributions plotted on a logarithmic scale to accommodate the wide range of unemployment rates across states. Bars indicate the share of users classified as unemployed in each group, and the overlaid black lines represent 95% CI. The benchmark (ground truth) distribution from official statistics is shown in gray bars. Inferences on gender and age are available for the 23 million users in our sample with a valid profile picture. JoblessBERT outperforms the rule-based model, both in terms of precision and recall of detecting unemployment self-disclosures, and in producing a sample that is more representative of the general US population.

First, we evaluate the ability of the two language models, namely JoblessBERT and the rule-based model, to detect disclosures of a user’s unemployment status on Twitter. We find that JoblessBERT considerably improves the classification of unemployment disclosures compared to the rule-based model (Fig. 1A). As one might expect, the rule-based model achieves a high level of precision (93.1%) with a relatively low recall (29.3%). In contrast, our JoblessBERT model retrieves nearly three times more relevant content about unemployment (recall of 76.5%; P<0.001) with the same level of precision. As shown in Fig. 1A, our model maintains high precision (>0.85) when retrieving more than 90% of the relevant disclosures. In other words, compared to existing methods, JoblessBERT not only finds more people talking about losing their job but also does so without introducing more false alarms. A closer examination of the linguistic patterns identified by JoblessBERT reveals that JoblessBERT expands beyond the frequent and intuitive patterns used in previous work (32). For example, JoblessBERT picks up expressions that contain spelling mistakes (“neeeeeed a job”) and slang (“needa job”), which are prevalent on social media but are unlikely to be preconceived. These differences also considerably expand the set of users whose expression is captured: JoblessBERT identifies nearly 13 times more unemployed users than the rule-based model. To make this distinction more concrete, we present illustrative examples of tweets captured by JoblessBERT but missed by the rule-based model in Fig. S4 (Supplementary material), which showcases JoblessBERT’s effectiveness in recognizing diverse forms of self-disclosure.

JoblessBERT also yields a more representative sample of users than the rule-based model. Examining the proportion of unemployed users in different states (Fig. 1B), we find that the rule-based model under-represents states where unemployment is low and over-represents states where unemployment is high: the slope of a fitted linear model yields a slope of 0.38, which is significantly different from an identity line (P<0.001). In contrast, JoblessBERT’s sample is more closely aligned with the actual distribution of unemployment across US states, having a regression slope of 0.86, which is not significantly different from an identity line (P>0.15). While this represents a substantial statistical improvement, it is important to note that for many individual states, the absolute differences between models are modest, and the log-scale presentation may visually amplify some of these distinctions (see Fig. S5 for linear-scale comparison). Figure 1C further shows that JoblessBERT demonstrates improved alignment with the official age distribution for some demographic groups, particularly users aged 20–29 and 30–39, where the proportions of unemployed users are not statistically different from official data. However, both models continue to over-represent unemployed youth (below 20), with JoblessBERT showing only modest improvement over the rule-based model in this age group. For users aged 40 and above, both approaches show similar performance with limited alignment to official statistics. The over-representation of younger users in both models reflects the underlying age skew of the Twitter user base in our sample (P<0.001, see Fig. S1). Finally, in terms of gender, Fig. 1D shows that the proportion of women unemployed users in JoblessBERT is closer to that of the official data than the rule-based model, although the proportions in both models are not statistically different from that of the official data (P>0.10). Taken together, these findings highlight that our fine-tuned Transformer-based classifier captures a broader variety of linguistic patterns describing unemployment, a substantially larger sample of users disclosing their unemployment status, and a sample that resembles more closely the official statistics of unemployment across states, age brackets, and genders. Additional robustness checks comparing users with and without valid geolocation information reveal only modest differences in unemployment disclosure rates (Fig. S6). Disclosure rates are not statistically different under the rule-based (P>0.10) and JoblessBERT models (P>0.10). These results indicate that heterogeneity in unemployment disclosure propensities is unlikely to be a primary driver of our main findings.

### Monitoring unemployment in real-time

**Fig. 2.**
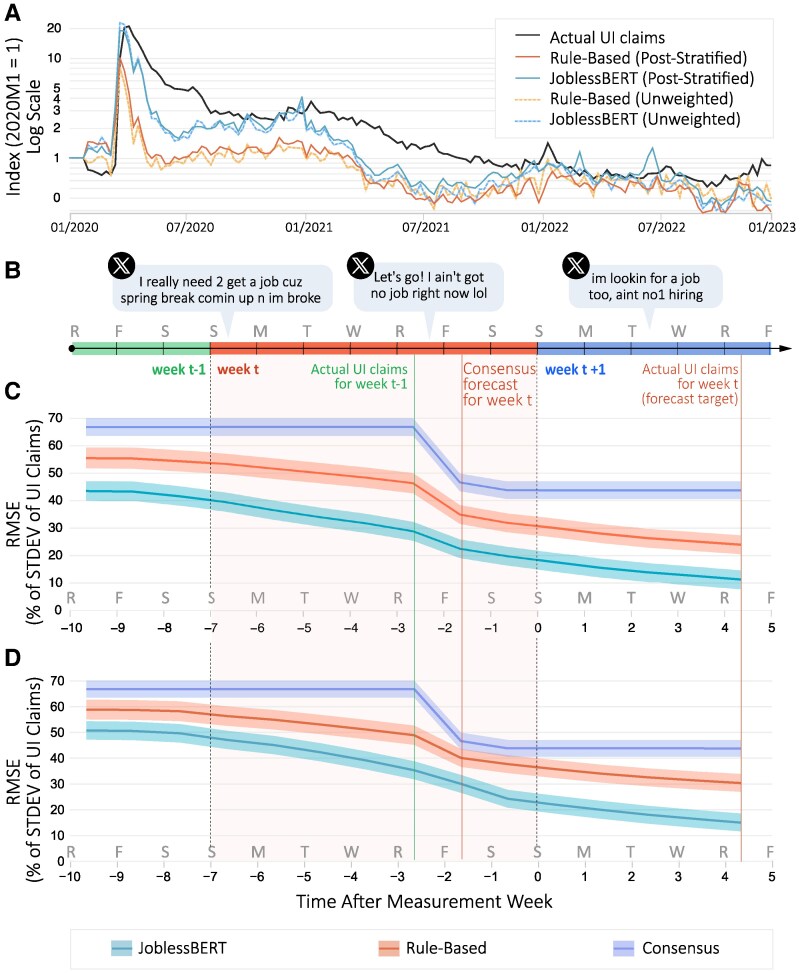
Predicting the US weekly initial claims for unemployment insurance (UI). A) Time series of unemployment indices derived from social media, plotted alongside actual UI claims on a log scale and normalized to January 2020 levels. B) Timeline of the real-time data flow of forecasting inputs relative to the end of the measurement week. C) National-level predictions of unemployment insurance claims in the United States using post-stratified versions of the social media index as a function of the forecasting horizon. D) National-level predictions of unemployment insurance claims in the United States using unweighted versions of the social media index. The figure compares three models: the baseline consensus model, the rule-based model, and the JoblessBERT model. The horizontal axis represents the number of days relative to the end of the measurement week (day 0), and vertical axis reflects forecast accuracy. Forecasting accuracy is measured in RMSE as a share of the SD of UI claims. Shaded bands around point estimates denote 95% CI.

Next, we investigate whether self-disclosures of unemployment on Twitter can help monitor UI claims. The numbers of UI claims for the current week—which ends on Sunday 12:00 AM—are published on Thursday 8:30 AM the following week. This lag of more than 4 days has created a space for an industry of professional forecasters, who publish their estimates almost 2 days before the work week ends, on Friday morning (Fig. 2B). In contrast, disclosures of unemployment by Twitter users, signaling potential eligibility for unemployment benefits, are available continuously throughout the week. Therefore, we construct daily estimates of weekly UI claims as the proportion of Twitter users who disclosed being unemployed in a 7-day sliding window out of all active users during the time window. We distinguish four types of such unemployment indices: unweighted indices, which are based on the raw numbers produced by the two language models (rule-based and JoblessBERT), and post-stratified indices, which are reweighted based on US census population estimates from the previous month and using inferred age, gender, and state information of users (see Methods for details).

Figure 2A shows the actual UI claims and the four indices constructed based on Twitter disclosures of unemployment on a logarithmic scale (10-based). Each series is normalized by its average value in the first month of January 2020, indicating, for instance, that UI claims rose by 20 orders of a magnitude 2 weeks after COVID-19 was declared a pandemic (on 2020 March 28) relative to actual claims recorded in January 2020. As shown in the figure, the unweighted indices tend to underestimate the magnitude of UI claim fluctuations, particularly during volatile months of the pandemic. However, they still capture the overall directional trends in unemployment reasonably well, including the timing and relative scale of key turning points. The post-stratified indices, by contrast, more closely align with the levels of actual UI claims across the entire period (P<0.001). We use the root mean squared error (RMSE) to measure how close our predictions come to the real UI claims numbers. A lower score means better predictions. The RMSE of JoblessBERT indices relative to UI claims are significantly lower than those of the rule-based indices (P<0.001), with the post-stratified JoblessBERT index significantly outperforming all other indices (P<0.001). While the gains of the post-stratified JoblessBERT index over the post-stratified rule-based index may seem small on a logarithmic scale, they are meaningful in absolute values. At the height of the pandemic (March to June 2020), the post-stratified rule-based index under-estimates UI claims, on average, by 54.5% more than the post-stratified JoblessBERT index (RMSE of 7.74 and 5.01 SD of UI claims, respectively), which translates to underestimation of 872,978 claims during this period (P<0.001). On an average week during the more stable times after June 2020, the differences between the post-stratified rule-based and post-stratified JoblessBERT indices are smaller (RMSE of 1.14 and 0.77 SD of UI claims, respectively), which translates to an average underestimation of 287,036 claims during this period (P<0.01).

Using the post-stratified Twitter indices, we next examine the ability of a dynamic model to predict UI claims up to 2 weeks in advance of the official data release. To identify any predictive gains from our Twitter-based indices, we consider three specifications of an autoregressive distributed lag model: (i) a baseline “consensus model” that only uses past UI claims releases and professional consensus forecasts, (ii) a “rule-based model” that adds to the baseline model the post-stratified rule-based Twitter index, and (iii) a “JoblessBERT model” that adds to the baseline model the post-stratified JoblessBERT Twitter index (see Methods for full model specifications). Figure 2C shows the RMSE of the three models as a function of time relative to the end of the measurement week. For example, the consensus model’s RMSE starts at 0.67 UI claims SD on day −10 (2 weeks before data release), drops to 0.46 SD on day −3 when the official release about the previous week becomes available, and reaches 0.43 SD after the consensus forecast is published on day −2. Across all three models, a lower RMSE is obtained as the release date draws closer, but there are important differences between models. First, there is a clear rank ordering between models, where on an average 2-week period before data release of actual UI claims (the forecast target), the rule-based index reduces the RMSE of the baseline model by 28.5%, and the JoblessBERT index reduces it by 54.3%. Moreover, the figure shows that the starting point for the JoblessBERT model 2 weeks ahead of the data release is on par with the performance of the baseline model at the end of the measurement week (d=0), when the baseline model has access to much more recent information.

While our primary analyses rely on post-stratified indices to account for demographic biases in the Twitter user base, it is important to assess the raw signal quality of each classification model. In Fig. 2D, we replicate the forecasting evaluation using unweighted indices, which are constructed directly from the raw share of users disclosing unemployment without applying post-stratification. We find that the unweighted JoblessBERT index continues to outperform the unweighted rule-based index across all forecast horizons. Over the 2-week forecast window, the JoblessBERT model using the unweighted index reduces RMSE by 38.6% relative to the baseline (P<0.001), compared to a 29.1% reduction for the unweighted rule-based model. Moreover, the unweighted JoblessBERT model’s accuracy is comparable to that of the post-stratified rule-based model, suggesting that JoblessBERT captures a substantially stronger raw signal from social media data. These findings highlight that the model’s gains are not solely driven by demographic reweighting, but by the JoblessBERT classifier’s ability to identify a broader and more meaningful set of unemployment disclosures.

Next, we examine whether the predictive strength of our Twitter signal depends on media coverage of unemployment, by partitioning our testing period into quartiles based on Google Trends composite scores for unemployment-related terms (see Section S8 for details). Although Google Trends reflects user search activity rather than direct media attention, search interest is often correlated with media attention, making it a useful proxy for periods when unemployment-related topics are more salient in public discourse. While forecast accuracy is highest during periods of elevated public attention (top quartile), the JoblessBERT model retains substantial predictive power even during low-attention periods, with RMSE increasing by only 15% when comparing the lowest to highest media attention quartiles (Fig. S8A). We also assess performance by year 2020–2022 and find that forecast accuracy peaks in 2020 amid heightened labor market disruptions (Fig. S8B). Nonetheless, JoblessBERT consistently outperforms rule-based and consensus forecasts across all years, demonstrating robustness to changing economic and platform conditions. Furthermore, when we test our models against an enhanced baseline that incorporates Google Trends unemployment indices (Fig. S9), JoblessBERT continues to provide significant forecasting improvements. These results suggest that our Twitter-based approach captures genuine labor market signals beyond what is reflected in user search activity related to unemployment.

It is also important to examine the model response to economic shocks. A pivotal example of such a shock occurred during the first week after COVID-19 was declared a pandemic (2020 March 14 to March 21), when UI claims jumped from about 252 thousand claims at the beginning of the week to 2.9 million claims at the end of it—an astounding increase of 4.1 SD (Fig. S7). We use this episode as a stress test to evaluate how different forecasting approaches responded in real time. The consensus model failed to anticipate the spike: using the industry’s estimate 2 days before the week ended, the model predicted only 327.2 thousand claims. On the same day, the rule-based model “sensed” the sudden change and predicted UI claims to reach 2.32 million, underestimating the true value by 20.5% and an improvement over the 88.8% underestimation of the consensus model. Finally, the JoblessBERT model forecasted 2.66 million UI claims 2 days before the week ended and 2.8 million claims on the day before the official release of 2.9 million. These results suggest that JoblessBERT could play a key role in an early warning system that senses changes in the labor market. Although this episode represents an extreme case, it illustrates the unique advantage of social media in rapidly sensing and adapting to sharp labor market disruptions when timely information is most needed. A subsample of users is sufficient to retain much of the predictive accuracy with a substantially smaller sample size (see Fig. S3 for details).

### Monitoring unemployment subnationally

Focusing on national trends may obfuscate large variability in unemployment across local labor markets (33). Tracking sub-national dynamics is critical for understanding spatial heterogeneities as they occur, especially during a crisis, and for designing place-based policies (34). Therefore, we evaluate the predictive performance of our models at the sub-national level by estimating a separate model for each US state and city. Since the consensus forecast is only available at the national level, we do not include it in our subnational models (see Methods).

**Fig. 3.**
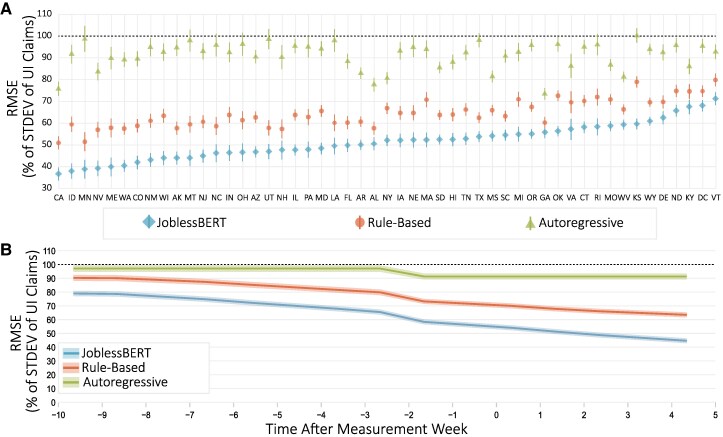
Sub-national predictions. A) RMSE of JoblessBERT, rule-based, and autoregressive models in predicting state-level unemployment insurance (UI) claims, shown separately for each state. B) RMSE of state-level forecasts as a function of the number of days relative to the end of the measurement week (day 0), comparing JoblessBERT, rule-based, and baseline autoregressive models. For all panels, RMSE is normalized by the SD of actual UI claims in the respective region. Shaded bands and vertical lines around point estimates denote 95% CI.

In line with the national-level results, we find that JoblessBERT robustly outperforms other models across all US states (Fig. 3A). On an average 2-week period before data release of actual UI claims, JoblessBERT’s predictions are 36.2% more accurate than the baseline (P<0.001) and 20.6% more accurate than the rule-based model (P<0.001). As shown in Fig. 3B, the JoblessBERT model yields substantial error reduction 2 weeks ahead of the state data release compared to the baseline’s prediction using all available information the day before the official release. It is also important to note that the accuracy of both the rule-based and JoblessBERT models steadily improve over time as more social media disclosures become available. To ensure these results are robust to baseline specification, we also compare against an enhanced baseline incorporating state-level Google Trends indices for unemployment-related search terms, which represents a more realistic forecasting approach available to practitioners (Fig. S10). Even against this stronger benchmark, JoblessBERT maintains significant predictive advantages, achieving 28.4% better accuracy (P<0.001).

Finally, JoblessBERT also outperforms other models at the city level, with accuracy improving in cities with higher Twitter use and more variable UI claims (Fig. S11A). To test the ability of our approach to compensate for gaps in official statistics, we evaluate the performance of the JoblessBERT model in ten “holdout cities,” where official UI claim numbers are rarely or irregularly updated (see Methods M8). As lagged variables are often missing in holdout cities, we substitute the city autoregressive terms in our models with state-level UI claims (see Methods). Figure S11C and D (as well as Fig. S11B) shows that forecast errors for holdout cities (hollow points) are comparable to those of cities with regularly updated UI claims (full points). These results indicate that the JoblessBERT predictions are valuable even when actual UI claim numbers are unavailable during training, suggesting that signals extracted from social media can fill gaps in traditional measures of unemployment at the city level.

## Discussion

This study demonstrates that a Transformer-based classifier fine-tuned to detect self-disclosures of unemployment on social media can serve as a leading indicator of unemployment in the United States. By combining a BERT-family model with Active Learning, we extract substantially more relevant content about unemployment than existing rule-based approaches, without sacrificing retrieval quality. The results also suggest that the additional content identified by the classifier is not merely a replication of the same linguistic patterns but rather a diverse set of expressions by a more representative sample of the target population. Post-stratification using inferred demographics of users considerably improves the alignment of our Twitter-based unemployment index with UI claims. Incorporating this index in a predictive model significantly enhances the accuracy of UI claim forecasts at national, state, and city levels up to 2 weeks before official data releases, outperforming professional forecasters, particularly during significant changes in unemployment.

The index can have several important applications. This work serves as a proof of concept demonstrating the feasibility of extracting timely, geographically granular economic signals from digital trace data. These insights could inform future collaborations between platforms and statistical agencies, support the development of more accessible data pipelines, and provide useful inputs for research and private-sector analysis. The index can also uncover measurement errors in official statistics: for example, during 1 week in May 2020, official UI claims in Connecticut reportedly increased sharply from 36,148 to 298,680, only to be corrected the following week to reflect a slight decrease to the level of 30,046 claims (8). The absence of such a spike in the JoblessBERT index could have assured government officials, and perhaps even the market, that the official measurement was off. Moreover, sensing changes in particular geographical locations using social media data could help surveyors decide to shift their samples to areas where changes are happening, leading to more accurate estimates and tighter error bounds. Finally, social protection agencies could use these social media indicators of unemployment to advertise training programs to relevant audiences and help connect job seekers with relevant opportunities.

More generally, the approach used in this work bears promise for other forecasting or estimation tasks that might benefit from the aggregation of public opinion. The general approach of iterative training of a Transformer encoder-based classifier to capture a more diverse set of linguistic forms can be applied to identify other rare forms of expression such as symptoms of a relatively rare disease or hateful and violent speech. The relatively simple adjustment and prediction methods used in this study provide an interpretable solution that facilitates the inspection of model outcomes and any anomalous predictions it may generate. As such, this modeling approach can potentially supplement additional estimation tasks in other fields including economics (e.g. inflation expectations, perception of policy-related outcomes), public health (e.g. rise in particular symptoms), politics (e.g. candidate support), environmental protection (e.g. climate change awareness), and more.

### Limitations and future directions

The current study uses simple aggregation methods to construct indicators and parsimonious linear models to construct predictions. Richer time series models with additional predictors and nonlinearities can further improve the predictions. As social media becomes progressively more prevalent, network effects could increase users’ incentives to signal their unemployment status (35, 36), decreasing forecast errors even further. Our work demonstrates the potential of social media-based unemployment indicators, but real-world implementation faces practical barriers, including demographic differences in disclosure norms, potential terms-of-service (ToS) constraints, and the technical demands of large-scale data collection, demographic inference, and geolocation processing. Post-stratification adjusts for representativeness in observable demographics but cannot fully correct for unobserved differences in disclosure norms across demographic groups. This represents a theoretical possibility that we cannot address directly, as we observe only users’ choice to disclose unemployment, not their true employment status. While our analysis suggests that group-level differences in observed disclosure rates are modest, this remains an important caveat when interpreting social media-based indicators.

This study focuses on English posts in the United States, but the same statistical approach can be applied to other languages, particularly local languages spoken in developing countries. The added value of our approach is potentially high in countries whose statistical agencies lack the resources to regularly collect reliable labor market data (4, 37). This approach could be replicated on other social media platforms with better coverage in these developing countries, such as Facebook. Extending this work to other countries could also provide valuable cross-national comparisons, shedding light on how unemployment self-disclosures vary across linguistic, cultural, and institutional contexts. However, success will depend on overcoming data access restrictions and ensuring the availability of reliable local validation sources.

Our findings provide a proof of concept: when available, social media data can provide timely, granular insights into economic conditions, supporting the development of scalable and accessible indicators. However, the broader social benefits of this approach depend on the continued access to large-scale user-generated content, and it remains an open question whether social media platforms will continue to provide such access. Since our data collection period (2020–2022), Twitter/X has undergone substantial changes under new ownership, including shifts in moderation practices, user demographics, and data access policies. In particular, access to Twitter’s data has become significantly more restricted since 2023, limiting the feasibility of replicating our methodology in real time. While this poses challenges, regulatory efforts such as the EU’s Digital Services Act may help restore or expand data availability for researchers and policymakers. It is also worth noting that our study period coincided with a wave of high-profile tech layoffs that likely amplified unemployment disclosures on social media. Nonetheless, our core finding that social media carries early labor market signals remains relevant. Going forward, model performance will depend on continuous adaptation to evolving platform conditions. Importantly, our framework of combining Transformer-based classification with Active Learning is platform-agnostic and can be applied to other sources of user-generated content, such as YouTube, TikTok, Bluesky, or Google Trends.

## Supplementary Material

pgaf309_Supplementary_Data

## Footnotes

a
https://huggingface.co/worldbank/jobless-bert
