# Supplementary material for: Can social media reliably estimate unemployment?
Source: PNAS Nexus. 2025 Dec 30;4(12):pgaf309. doi: 10.1093/pnasnexus/pgaf309 (PMC12750448; doi:10.1093/pnasnexus/pgaf309)
Supplement: pgaf309_Supplementary_Data [file pgaf309_supplementary_data.pdf]

Supplementary Information for
Can Social Media Reliably Estimate Unemployment?

Do Lee, Manuel Tonneau, Boris Sobol,
Nir Grinberg, and Samuel P. Fraiberger*

*Corresponding author; E-mail: sfraiberger@worldbank.org.

The PDF file includes:

Supplementary Methods

Supplementary Text

Figs. S1 to S11

References *(37-44)*

Supplementary Methods

M1. Unemployment Insurance (UI) claims data.

The dataset on unemployment comes from weekly releases of the initial claims for unemployment insurance (millions, not seasonally adjusted) between 2016 and 2022. National and state level UI claims for a given week are released by the U.S. Department of Labor (DOL) at 8:30 AM (Eastern) each Thursday of the following week. The UI claims for a given week cover the total number of claims filed during the week, where the observation week ends on Sunday at 12:00 AM. The statistic is also publicly available for selected cities through the Opportunity Insights Economic Tracker (*6*) and the state DOL websites that publish the data. We include in the analysis cities whose UI claims data is available for at least one year between 2016 and 2022.

M2. Consensus forecast data.

The dataset on consensus professional forecasts comes from Bloomberg (*27*). We use the median forecast from a survey of around 50 economists and the forecasts are observed as of 8:30 AM on the Friday of the week prior to the UI data release. The forecast target is the UI claims for the current week, which is scheduled to be released the following Thursday.

M3. Twitter data.

Our Twitter dataset is collected using the Twitter Application Programming Interface (API). We start from the Twitter Decahose – a 10% random sample of all tweets – and then conduct country location inference to identify US-based users. Specifically, we use the Google Geocoding API as it was shown to be highly accurate in past work (*37, 38*) and apply it on self-reported user locations to map each user to a country. We then restrict the analysis to users with an inferred profile location in the U.S. and collect all their tweets and those of the users they men-

tioned. We drop retweets as we aim to detect self-disclosures of employment status. We also drop near-duplicate tweets associated with holiday cash giveaways because they are one-time events that can create false spikes in the number of unemployed-related tweets. In total, we gather a dataset of about 15.7 billion tweets posted between January 2009 and December 2022 by 31.5 million users with a profile location in the U.S.

User-level data include their profile information from which we inferred their age brackets, gender (binary), city and state (Supplementary Fig. S1). The four age brackets are 20 years old and below, 20 to 29, 30 to 39, 40 and above. We use *m3inference* (*39*), a deep-learning model for Twitter user demographic inference, to predict the age and gender of users in our sample. Based on prior validation studies (*39*), *m3inference* achieves approximately 90.7% macro-F1 score for binary gender classification and 52.2% macro-F1 score across coarse age brackets. We are only able to infer the demographics of users with a profile picture, which represent 67 percent of all users. We include all users with valid demographic inferences in our post-stratification, and impute missing attributes by sampling from the observed demographic distribution (see Materials and Methods M6).

M4. Language models.

We consider two alternative language models to classify disclosures of unemployment status in tweets' textual content. Both models focus on self-disclosures to avoid double-counting a user posting about the unemployment status of another user.

The first is a rule-based model similar to the one used in previous work (*19*): starting from a handpicked list of 75 highly specific sentences – e.g., "I just lost my job" – we classified a user as disclosing their unemployment status if they posted a tweet which matches one of the keyphrases in the list. Supplementary Fig. S2 shows all the keyphrases used in the rule-based model and the share of tweets that matches each pattern.

The second is a large language model (LLM) we coin *JoblessBERT*. It is based on Conversational BERT (*29*), an encoder-based large language model pre-trained on large amounts of unlabeled social media text to learn latent word representations conditioned on the context in which they appear in tweets. To develop JoblessBERT, we work with all tweets from the Twitter dataset posted before December 2020 and we sampled tweets posted by US-based Twitter users containing job-related motifs. The full list of motifs is: "(i, unemployed)", "unemployed", "(i, jobless)", "jobless", "unemployment", "(i, fired)", "i got fired", "just got fired", "laid off", "lost my job", "(anyone, hiring)", "(wish, job)", "(need, job)", "(searching, job)", "(looking, gig)", "(applying, position)", "(find, job)", "(found, job)", "(just, hired)", "i got hired", "(got, job)", "new job", "job", "hiring", "opportunity", "apply". The use of parentheses indicate regular expressions matching all strings containing the words in the parentheses in the order in which they are indicated. We provide more details on the selection of motifs in (*28*). We then task Amazon Mechanical Turk crowdworkers to annotate a sample of these tweets to indicate whether they believe the author is unemployed or not, yielding an annotated stratified sample of 4,506 tweets. Using this sample, we then fine-tune Conversational BERT to the binary classification task of characterizing a tweet's author as unemployed or not. Subsequently, we use active learning, which is a sampling approach aiming to retrieve the most informative instances in order to maximize classification performance for a given annotation budget (*26*). Specifically, we develop an iterative active learning approach called *exploit-explore retrieval* which aims to maximize precision while improving recall by feeding new and diverse instances at each iteration. In total, we run 13 iterations of exploit-explore retrieval until exhaustion of our annotation budget, yielding an additional 4,332 annotated examples. The crowdsourced annotation was conducted between January and July 2021. Finally, we finetune Conversational BERT for the same binary classification task using all 8,838 annotated examples, yielding the JoblessBERT model we use in this study. We note that of all the tweets sent to annotation, we obtained ma-

jority agreement among annotators for 95% of tweets and we only kept tweets with majority agreement for training. We provide more information on the annotation task and the active learning approach in (*28*). We also open-source JoblessBERT on Hugging Face under this link: `https://huggingface.co/worldbank/jobless-bert`.

It is important to note that JoblessBERT attributes a low score when a user is not talking about their own unemployment status. For example, users may comment general news about the unemployment rate, as in the tweets "so sad how many people are losing their jobs" and "crazy how high the unemployment rate is". In these cases, JoblessBERT assigns a score of 0.0009 and 0.01 respectively. Users may also mention their past unemployment (e.g., "I used to be unemployed but I now finally found a new job") or the unemployment of somebody else (e.g., "My brother doesn't have a job"). In these cases, JoblessBERT also assigns a low confidence score of 0.13 and 0.23 respectively. Finally, users may think that they are fired even though they are not (e.g., "I thought my manager would fire me when I got to work today, but thankfully that didn't happen"). In this case, JoblessBERT assigns a confidence score of 0.0. In contrast, the classifier successfully assigns high scores to tweets revealing its author's unemployment status, such as 0.9946 to "I am unemployed", 0.99 to "I don't have a job" and 0.99 to "My manager fired me when I got to work today".

M5. Twitter unemployment index.

We construct a daily unemployment index as the proportion of active users disclosing their unemployment status as follows:

$$u^m_{t,d} = \frac{U^m_{t,d}}{N_{t,d}}, \tag{1}$$

where t represents a UI week (ending on Sunday 12:00 AM). d indexes the number of days relative to the end of the UI week t. $U^m_{t,d}$ is the number of users disclosing their unemployment status according to language model m over a trailing 7-day window that ends on day d. $N_{t,d}$ is

the number of active users over the same trailing 7-day window ending on day d, where active users are defined as users that posted at least one tweet in this trailing window.

M6. Post-stratification.

To adjust for a lack of representativeness along observed dimensions, we reweight our sample of active users by age, gender and state based on data from the U.S. Census Bureau from the previous month. The post-stratified unemployment index is defined as follows:

$$\tilde{u}^m_{t,d} = \frac{\sum_{(a,g,s)\in\{A,G,S\}} L_{a,g,s,t,d} \times \frac{U^m_{a,g,s,t,d}}{N_{a,g,s,t,d}}}{\sum_{(a,g,s)\in\{A,G,S\}} L_{a,g,s,t,d}} \tag{2}$$

where t represents an unemployment insurance week (ending on Sunday 12:00 AM). d indexes the number of days relative to the end of the week t. $U^m_{a,g,s,t,d}$ is the number of users in demographic group (a, g, s) disclosing their unemployment status according to language model m during a seven-day period ending on day d. $N_{a,g,s,t,d}$ is the number of active users in demographic group (a, g, s) during the same seven-day period. $L_{a,g,s,t,d}$ is the size of the labor force in demographic group (a, g, s) during the previous month in the Census data.

We create the demographic group (a, g, s) by considering all possible combinations of age (4 categories), gender (2 categories), and state (51 categories), leading to a partition of the data into 408 cells. Gender groups G consist of either male or female. Age brackets A are 20 years old or below, 20 to 29, 30 to 39, or 40 and above. When user demographics are not available, we impute the missing values by sampling randomly from the distribution of users with available demographic inferences. Data for the size of the labor force $L_{a,g,s,t,d}$ come from the population estimates of the U.S. Census Bureau. The number of users in each demographic group $N_{a,g,s,t,d}$ is based on inferences made from users' profile.

Post-stratified unemployment indices at the state level are constructed in a similar fashion by computing equation (2) using a demographic partition by age, gender, and city. For city-level indices, the user sample is simply partitioned by age and gender.

M7. Predictive model (national).

Our model incorporates the most recent information in three periods: before the release of the previous week's UI claim numbers ($-10 \leq d < -3$), before the release of professional forecasters estimates ($-3 \leq d < -2$), and after the forecasters' release on $d = -2$ and before the official release on $d = 4$. We explore the ability of the unemployment index to predict UI claims two weeks ahead of the current release using the following autoregressive distributed lag model:

$$\hat{y}_{t,d} = c + \sum_{i=d}^{d-6} \theta_i \tilde{u}^m_{t,i} + \begin{cases} \alpha_0 y_{t-2} + \alpha_1 y_{t-3} + \cdots + \alpha_p y_{t-2-p} & \text{if } -10 \leq d < -3 \\ \beta_0 y_{t-1} + \beta_1 y_{t-2} + \cdots + \beta_p y_{t-1-p} & \text{if } -3 \leq d < -2 \\ \gamma f_t + \beta_0 y_{t-1} + \beta_1 y_{t-2} + \cdots + \beta_p y_{t-1-p} & \text{if } -2 \leq d < 4 \end{cases} \tag{3}$$

where time t is measured in weekly intervals and represents an unemployment insurance week (ending on Sunday 12:00 AM). d indexes the number of days relative to the end of the week t. $\hat{y}_{t,d}$ is the predicted UI claims for week t based on information observed up to day d. y_t is the actual UI claims for week t. $\tilde{u}^m_{t,d}$ is the unemployment index according to language model m. f_t is the consensus forecast. Both y_t and f_t are normalized by the labor force during the previous month. We measure the size of the labor force using the civilian noninstitutional population estimates for the U.S. from the Bureau of Labor Statistics. p denotes the maximum number of autoregressive lags. c is the intercept. $\theta_d, \ldots, \theta_{d-6}$ are coefficients on the unemployment index. γ is the coefficient on the contemporaneous value of the consensus forecast. $\alpha_0, \ldots, \alpha_p$ are coefficients on lags of UI claims before y_{t-1} is released. $\beta_0, \ldots, \beta_p$ are coefficients on lags of UI claims after y_{t-1} is released.

For each specification, we combine the estimated parameters with input data to form predictions. Forecasting performance is measured in terms of the Root Mean Squared Error (RMSE) averaged over the test period of 2020 week 1 to 2022 week 52. To form predictions for 2020 week 1, the data used to train and validate the autoregressive model covers the period from 2016 week 1 to 2019 week 52. To account for time variations in the model parameters, we re-

estimate them each week as new information becomes available using a training sample whose observations lie within a backward-looking 4-year rolling window (*40*).

To quantify the ability of the unemployment index to track UI claims in near real-time, we evaluate the RMSE over a range of forecast horizons, starting from the official UI claims release two weeks ahead of the current release (i.e. $d = -10$), and up to the day prior to the current release (Supplementary Fig. 2A). All the predictions only use observable information available at the time of the forecast (Supplementary Fig. 2B): for example, as y_{t-1} is released on Thursday of week t, we only include it in the model when $-3 \leq d < 4$. Similarly, as f_t is released on Friday of week t, we only include it in the model when $-2 \leq d < 4$.[1]

To highlight the predictive gains resulting from our unemployment index, we then compare three specifications for equation (3): (i) the baseline "consensus model" where we set $\theta_i = 0$, (ii) the "rule-based model" where $m =$ *rule-based*, and (iii) the "JoblessBERT model" where $m =$ *JoblessBERT*.

To account for the unprecedented shock and government response at the onset of the COVID-19 pandemic (*8*), we allow the estimated parameters to shift between two regimes indexed by the state variable $s_t \in \{0, 1\}$, which is equal to one during the period going from 2020 week 9 through 2020 week 22 and zero otherwise (*41*). The COVID-19 regime starts from the week of the COVID-19 disaster declaration in the U.S. and covers the early phase of the pandemic. During this period, the federal and state governments expanded its unemployment insurance benefits to unprecedented levels through the Federal Pandemic Unemployment Compensation (FPUC) and Pandemic Emergency Unemployment Compensation (PEUC) programs (*8*). To ensure that the predictive model uses only information available in real time, we introduce the state variable s_t only for models estimated after 2020 week 22, after the COVID-19 regime has been fully realized.

[1]Note that since both the official and professional estimates are released at 8:30 AM, the entire time series is anchored at this time and not midnight, i.e., $d = 0$ corresponds to Sunday at 8:30 AM.

M8. Predictive model (sub-national).

We compare the state-level and city-level predictions from three specifications for equation (3): autoregressive, rule-based, and JoblessBERT. Unlike for the country-level predictions, we do not compare the rule-based and LLM specifications against the consensus model at the state and city level because consensus professional forecasts are not available at these levels of granularity. For this reason, we use an autoregressive model as our baseline model and no longer include the consensus forecast as one of the predictors in equation (3).

The parameters for each predictive model are estimated separately for each state and city. Each state- or city-level model is the same as the corresponding national model, but with additional predictors relevant to the state or city being predicted. To predict UI claims for a state, we use lags of UI claims and unemployment indices at the national level and for the state being predicted. To predict a city's UI claims, we use lags of UI claims and unemployment indices at the national level, the largest state onto which the city expands to, and the city being predicted.

For the city-level predictions, we divide cities into two groups based on the availability of ground truth data on UI claims. Trained cities are cities with at least one year of data available before 2020 week 1 to estimate the predictive model and whose UI claims data are released regularly on a weekly basis at 8:30 AM (Eastern). The list of trained cities are: Atlanta, Beaumont, Bloomington, Boise, Boston, Cedar Rapids, Chicago, Cleveland, Columbus, Corpus Christi, Dallas, Des Moines, Dubuque, Fort Wayne, Honolulu, Houston, Idaho Falls, Kansas City, Killeen, Lafayette, Las Vegas, Longview, Lubbock, McAllen, Midland, Laredo, Reno, San Angelo, Seattle, Sioux City, South Bend, Springfield, Waco, Washington D.C., and Waterloo.

We set aside a separate set of holdout cities that are not used to estimate the parameters of the predictive model. The holdout cities are ones whose UI claims data releases are less frequent than weekly. Predictions for each holdout city are based on parameters estimated for the city's state together with the city's input data. The holdout cities are: Abilene, Amarillo, Austin,

Brownsville, College Station, Davenport, Hartford, Indianapolis, San Antonio, and Wichita Falls.

M9. Cross-validation.

We estimate the hyper-parameters of the predictive model using cross-validation. The cross-validated parameters are: (i) the order of the lag polynomial $0 \leq p \leq 4$, and (ii) the classification threshold of JoblessBERT $b \in [0.5, 0.995]$, which balances precision and recall (*28*). We hold out a test sample which spans 2020 week 1 to 2022 week 52. We cross-validate the hyper-parameters by applying the following steps:

1. We start by predicting UI claims for the first period of the test sample (2020 week 1) using information available up to t = 2020 week 1, day $d = -10$, where t represents an unemployment insurance week (2020 week 1 ending Sunday 12:00 AM) and d indexes the number of days relative to the end of week t.

2. We prepare a prior observation sample of size $N = 208$ weeks from $t-N$ = 2016 week 1, day $d = -10$ to $t - 1$ = 2019 week 52, day $d = -10$.

3. The observation sample is partitioned into two subsample windows: First, we prepare a training subsample consisting of the first $N_T = 156$ weeks from $t - N$ = 2016 week 1, day $d = -10$ to $t - N_V - 1$ = 2018 week 52, day $d = -10$. Second, we hold out a validation subsample of $N_V = 52$ subsequent weeks from $t - N_V$ = 2019 week 1, day $d = -10$ to $t - 1$ = 2019 week 52, day $d = -10$.

4. For each combination of hyperparameters p and b, we evaluate the model's performance by calculating the RMSE over pseudo out-of-sample forecast errors generated from rolling regressions through the validation sample. We select the hyperparameters $\widehat{p}, \widehat{b}$ that mini-

mize the pseudo out-of-sample RMSE:

$$\widehat{p}, \widehat{b} = \underset{p,b}{\operatorname{argmin}} \frac{1}{N_V} \sum_{\tau=t-N_V}^{t-1} \left(y_\tau - \hat{y}_{\tau,d}(\widehat{\mathbf{\Theta}}_{\tau,d,p,b}) \right)^2 \tag{4}$$

where $\tau = t - N_V, \ldots, t-1$ indexes the weeks over the validation subsample from 2019 week 1 to 2019 week 52. y_τ denotes actual UI claims for week τ which is scheduled to be released at 8:30 AM on Thursday of the following week. $\hat{y}_{\tau,d}(\widehat{\mathbf{\Theta}}_{\tau,d,p,b})$ is the forecast of y_τ based on the autoregressive distributed lag model in equation (3), where the parameters $\mathbf{\Theta}_{\tau,d,p,b} \equiv (c, (\theta_i)_{i=d}^{d-6}, (\alpha_i, \beta_i)_{i=0}^{p}, \gamma)$ were estimated given hyper-parameters p, b and data from a training sample of size N_T from week $\tau - N_T$, day d to week τ, day d:

$$\widehat{\mathbf{\Theta}}_{\tau,d,p,b} = \underset{\mathbf{\Theta}_{\tau,d,p,b}}{\operatorname{argmin}} \frac{1}{N_T} \sum_{s=\tau-N_T}^{\tau} \left(y_s - \hat{y}_{s,d}(\mathbf{\Theta}_{\tau,d,p,b}) \right)^2 \tag{5}$$

The time subscripts τ, d on $\widehat{\mathbf{\Theta}}_{\tau,d,p,b}$ are used to denote one in a sequence of time-invariant parameter estimates obtained from rolling training subsamples whose observations end on week τ, day d, rather than estimates that vary over time within a given sample.

5. Given the cross-validated hyper-parameters $\widehat{p}, \widehat{b}$ from equation (4), we make a prediction of y_t by using $\hat{y}_{t,d}(\widehat{\mathbf{\Theta}}_{t,d,\widehat{p},\widehat{b}})$, where the prediction combines the input data as of week t = 2020 week 1, day $d = -10$ with parameters $\widehat{\mathbf{\Theta}}_{t,d,\widehat{p},\widehat{b}}$ that are estimated given cross-validated hyper-parameters $\widehat{p}, \widehat{b}$ and data from a training sample of size N_T from week $t - N_T$, day $d = -10$ to week t, day $d = -10$.

6. Once steps 1 to 5 have completed, we roll the prior observation sample forward by one day from day $d = -10$ to day $d + 1 = -9$. The new observation sample covers $N = 208$ weeks from $t - N$ = 2016 week 1, day $d = -9$ to $t - 1$ = 2019 week 52, day $d = -9$. We repeat steps 1 to 5 listed above until we reach the day prior to the UI claims release, on day $d = 3$. We repeat the steps while keeping the same hyper-parameters $\widehat{p}, \widehat{b}$ that were selected based on equation (4) using information up to week t, day $d = -10$.

7. Once steps 1 to 6 have completed, we roll the forecast target forward by one week, from week t to week $t+1$. We repeat steps 1 to 6 to predict y_{t+1}, actual UI claims for week $t+1$, while using information available up to week $t+1$, day $d = -10$. We repeat the steps listed above until the last forecast is made for y_T, where $T = 2022$ week 52 is the last week of the test period.

8. For each forecast horizon indexed by day d, we report the unweighted average RMSE across the test periods from 2020 week 1 to 2022 week 52 as a share of the standard deviation of actual UI claims over the same period.

The optimal lag order chosen from this procedure is typically around 4 weeks and is similar across the alternative models we consider. The cross-validated cutoff scores lie within the interior of the range we consider.

To formally compare the predictive performance of competing models, we use Diebold-Mariano tests with a 5% confidence level (*42*). The 95% confidence interval CI of the Root Mean Square Error $\sqrt{\overline{e^2}}$ is:

$$CI = \left[\sqrt{\overline{e^2} - 1.96 \times \sigma^2_{\overline{e^2}}}, \sqrt{\overline{e^2} + 1.96 \times \sigma^2_{\overline{e^2}}}\right]$$

$$\sigma_{\overline{e^2}} = \sqrt{\frac{\gamma_0 + 2\sum_{k=1}^{\lceil n^{1/3} \rceil} \gamma_k}{n}}$$

$$\gamma_k = \frac{1}{n} \sum_{t=k+1}^{n} (e_t^2 - \overline{e^2})(e_{t-k}^2 - \overline{e^2})$$

where $\overline{e^2}$ is the mean-squared error over the test period and $\sigma_{\overline{e^2}}$ is the standard error of the mean-squared error. n is the number of observations across the test period, e_t is the prediction error at time t, and γ_k is the autocovariance of e_t^2 at lag k weeks. The autocovariances γ_k correct for serial correlation across periods. We summarize the relative performance of each model by expressing the RMSE of its prediction as a ratio relative to the standard deviation of UI claims. The standard error for the ratio is calculated using the Delta method.

Supplementary Text

S1. Summary statistics.

Supplementary Fig. S1 presents summary statistics that describe the demographic and behavioral features of Twitter users classified as unemployed in our sample. Panels A through D assess the representativeness of the sample by comparing the geographic and demographic distribution of users to official benchmarks. Panel A shows the distribution of unemployed users across U.S. states compared to the civilian labor force; the data are plotted on a logarithmic scale to accommodate wide variation in state-level unemployment counts. Panel B compares the same user distribution to aggregate personal income by state. Panels C and D compare the distributions of users by age and gender to their respective distributions in the labor force. Bars in these panels indicate the share of users classified as unemployed in each group, with overlaid black lines denoting 95% confidence intervals. The benchmark distributions from official statistics are shown in black bars. Demographic inferences for age and gender are available for the 23 million users in our sample with valid profile pictures. Panels E and F characterize user-level disclosure behavior. Panel E shows the distribution of the number of days between consecutive unemployment-related tweets per user, while Panel F shows the number of unemployment-related tweets per user. In both cases, we compare output from the rule-based model and JoblessBERT. These patterns highlight the increased expressiveness and disclosure coverage captured by JoblessBERT, which identifies more users with repeated unemployment-related posts and better reflects the persistence of job loss over time.

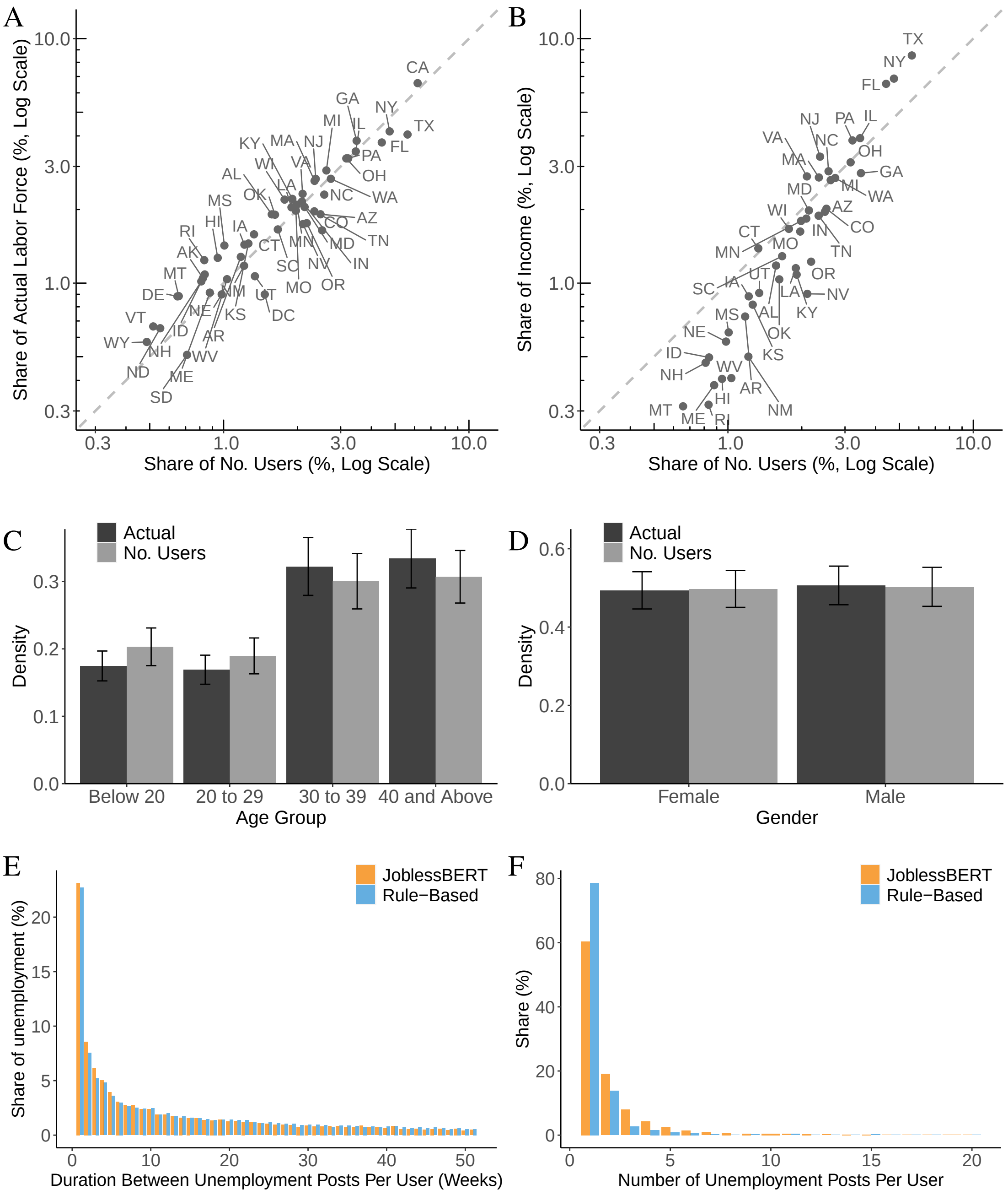

Supplementary Fig. S1: Summary Statistics.

(A) Distribution of users and distribution of actual labor force by state. **(B)** Distribution of users and distribution of personal income by state. **(C)** Distribution of users and distribution of actual labor force by age bracket. **(D)** Distribution of users and distribution of actual labor force by gender. **(E)** Duration between unemployment posts per user. **(F)** Number of unemployment posts per user.

S2. Distribution of the rules in the rule-based index.

Supplementary Fig. S2 presents the full set of hand-crafted phrase rules used in the rule-based model to identify unemployment disclosures on Twitter, and shows how frequently each phrase appears in the classified tweet sample. Each phrase (e.g., "I just lost my job") is treated as a binary rule: if a tweet contains one of these phrases, the user is classified as unemployed. The phrases were originally selected based on their specificity and face-validity in expressing personal job loss, following the approach in prior work (*19*). The figure plots the prevalence of each rule as the number of matched tweets divided by the total number of tweets that the rule-based model classified as unemployment disclosures. The x-axis is plotted on a logarithmic scale to accommodate the wide range of frequencies across rules. The resulting distribution is highly skewed: a small number of common expressions account for a large share of matched tweets, while many of the 75 phrases are rarely used. This pattern illustrates a key limitation of the rule-based model: it captures only a narrow slice of the linguistic variation people use to discuss job loss, often missing misspellings, slang, or paraphrased statements that would be captured by a learned model such as JoblessBERT.

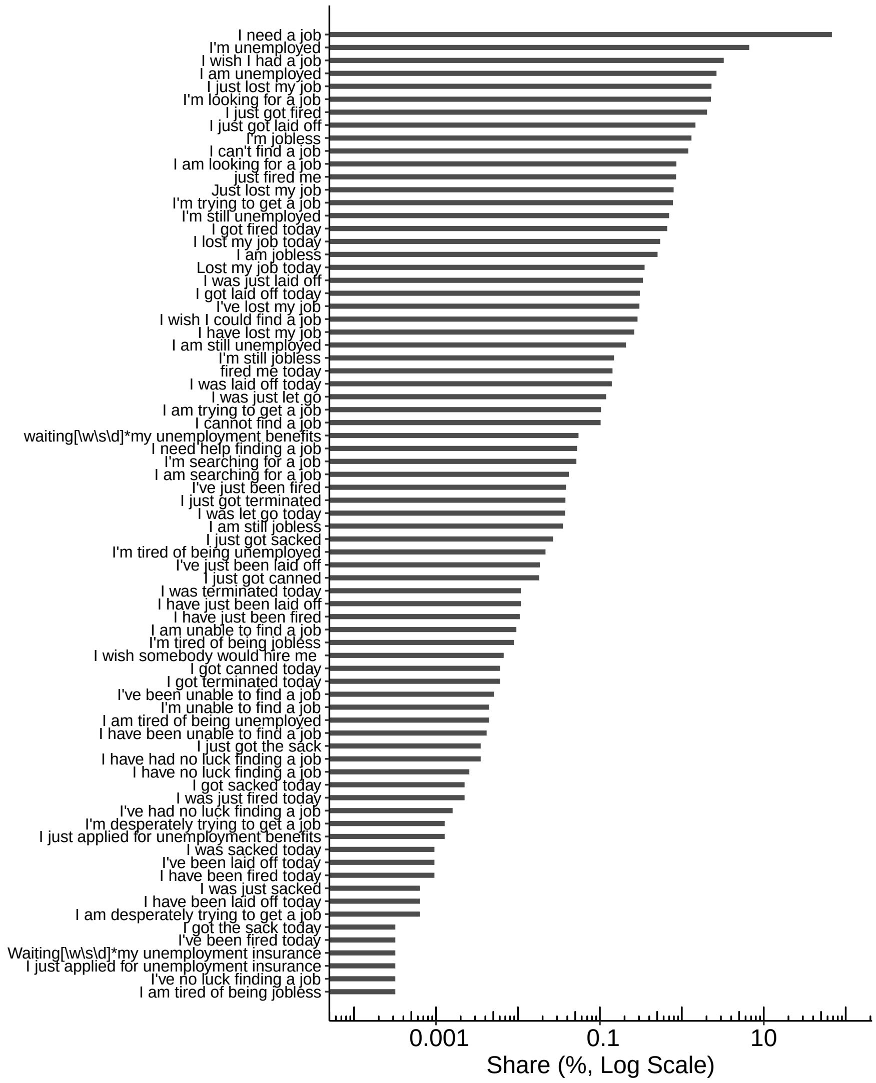

Supplementary Fig. S2: Distribution of the number of tweets for each rule in the rule-based index.

Each bar represents the share of matched tweets corresponding to a specific phrase used in the rule-based model. The x-axis is plotted on a logarithmic scale.

S3. Forecast Accuracy Under Alternative Downsampling Strategies.

While the main findings are obtained by collecting a very large sample of Twitter users covering almost 10% of the U.S. population, not all users are equally informative about unemployment trends. As social media data are becoming increasingly difficult to access (*43*), it begs the question of how to efficiently sample users in order to retain high predictive power while minimizing the amount of data being collected.

We consider two alternative user sampling schemes: (i) uniform and (ii) based on user's likelihood of disclosing their unemployment status. For each sampling scheme, we iteratively re-estimate equation (3) after having removed an increasing larger share of users. We fit a logistic regression to estimate the likelihood of a user disclosing their unemployment status (*44*):

$$P[u_{i,m} = 1] = logit^{-1}[\alpha_0 + a^{gender}_{j(i)} + a^{age}_{j(i)} + a^{state}_{j(i)} + \alpha_1 \ln(\text{Friends}_i) + \alpha_2 \ln(\text{Statuses}_i)]$$

where i indexes the user and $u_{i,m}$ is an indicator variable equal to one if the user is classified as unemployed based on language model m. α_0 is the intercept, and $a^{g}_{j(i)}$ denotes the coefficients for each group j in the categorical variable $g \in \{gender, age, state\}$. Gender denotes either male or female. Users are mapped into four age brackets from 20 years old or below, 20 to 29, 30 to 39, 40 and above. Friends_i and Statuses_i denotes the number of friends and statuses posted by user i, respectively.

We discover that by only sampling half of the users with the highest likelihood of disclosing their unemployment status, the JoblessBERT model's predictions averaged over a two-week period prior to the release date only deteriorate by 13.1%, compared to 44.3% with uniform downsampling (Supplementary Fig. S3). These results suggest that an efficient sampling strategy is to focus on users with a high likelihood of disclosing their unemployment status, enabling the JoblessBERT model to retain most of its predictive power while reducing the sampling cost by a factor of two.

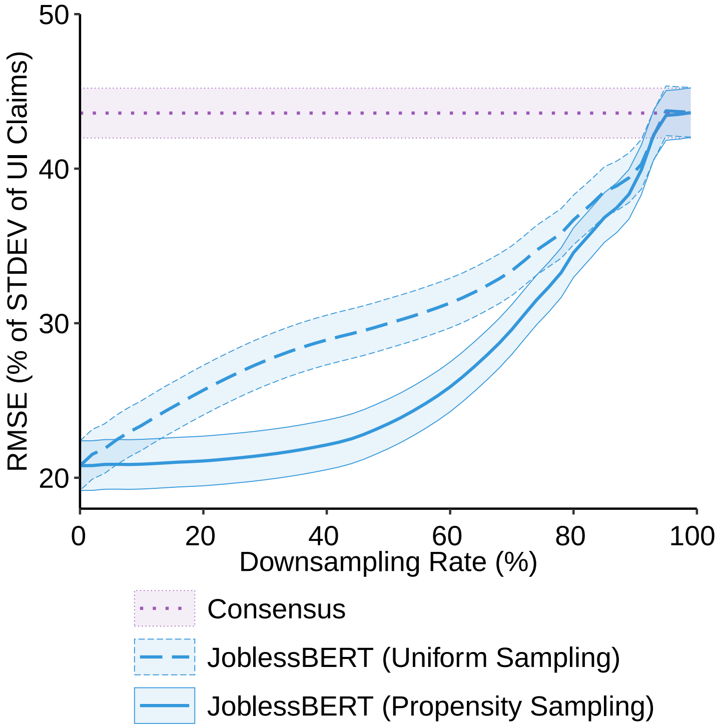

Supplementary Fig. S3: Forecast Accuracy Under Alternative Downsampling Strategies.
(A) Forecast accuracy of the JoblessBERT model under two downsampling strategies: uniform (random) and selective (based on predicted likelihood of unemployment disclosure). The x-axis shows the fraction of users retained, and the y-axis shows RMSE of national-level UI claim forecasts, averaged over a two-week period prior to release. RMSE is normalized by the standard deviation of actual UI claims. The JoblessBERT model is benchmarked against the Consensus forecast. RMSE is again normalized by the standard deviation of actual UI claims. Shaded bands around point estimates denote 95% confidence intervals.

S4. Examples of unemployment-related tweets identified by JoblessBERT.

To illustrate JoblessBERT's expanded linguistic coverage compared to traditional keyword-based approaches, we present representative examples of unemployment-related tweets that JoblessBERT successfully identified but the rule-based model missed entirely. These examples demonstrate how JoblessBERT captures the diverse and non-standard language patterns prevalent on social media platforms, including spelling variations, informal contractions, slang expressions, and social media abbreviations that are unlikely to be anticipated in predefined keyword lists. This broader linguistic variety contributes to JoblessBERT's ability to identify nearly 13 times more unemployed users than the rule-based model, significantly expanding the coverage of unemployment disclosures captured from social media data.

**Tweet Example**	**JoblessBERT**	**Rule-Based**
Spelling Variations & Misspellings		
"neeeeeed a job so badly right now 😭"	✓	✗
"unemployed and strugling to pay bills"	✓	✗
"desssperately looking for work"	✓	✗
Informal Contractions & Slang		
"needa job ASAP, bills are piling up"	✓	✗
"gotta find work before rent is due"	✓	✗
"tryna get hired somewhere, anywhere 🙏"	✓	✗
"they cut me loose. back on the market"	✓	✗
Social Media Abbreviations		
"OMG need a job rn, this is stressful 😰"	✓	✗

Supplementary Fig. S4: Examples of unemployment-related tweets identified by JoblessBERT but missed by the rule-based model.

The table demonstrates JoblessBERT's ability to capture non-standard language patterns including spelling variations and misspellings (e.g., "neeeeeed," "strugling"), informal contractions (e.g., "needa"), and social media abbreviations that keyword-based approaches cannot detect. ✓ indicates successful identification of unemployment disclosure; ✗ indicates missed detection.

S5. Distribution of unemployed users by state: Linear scale.

Supplementary Fig. S5 presents the same comparison as Fig. 1B, showing the distribution of unemployed users across U.S. states for both JoblessBERT and the rule-based model, but plotted on linear rather than logarithmic scales. Panel A displays the full range of state-level data points, while Panel B zooms in on the lower end of the distribution to highlight differences among smaller states with lower unemployment levels. The linear scaling provides a more transparent view of absolute differences between model estimates and the benchmark distribution.

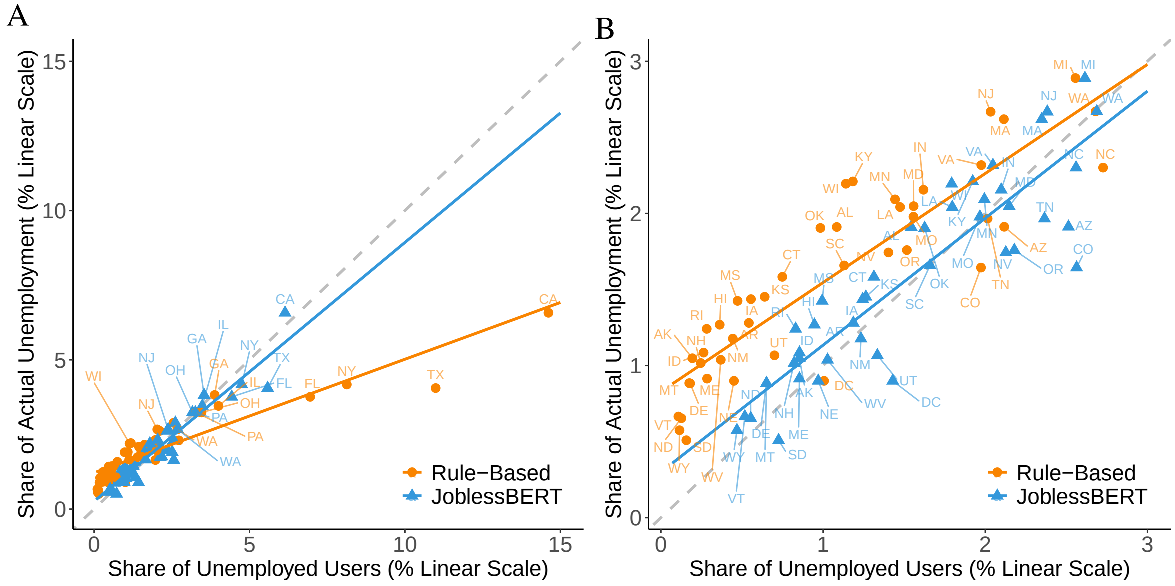

Supplementary Fig. S5: Distribution of unemployed users by state: Linear scale.
(A) Full range comparison showing all state-level data points plotted on linear scales. **(B)** Zoomed view focusing on the region near zero to highlight the magnitude of differences for smaller states with lower unemployment levels. This figure presents the same data as Fig. 1B using linear scales to provide transparency regarding absolute differences in the distribution of unemployed users between JoblessBERT and rule-based indices.

S6. Propensity of Unemployment Disclosure by Users With and Without Geolocation.

Supplementary Fig. S6 compares the unemployment status disclosure propensity between users with and without valid geolocation information. Disclosure propensity is defined as the share of a user's tweets that are classified as unemployment-related. The figure shows that the average propensity is similar across the two groups for both rule-based and JoblessBERT models.

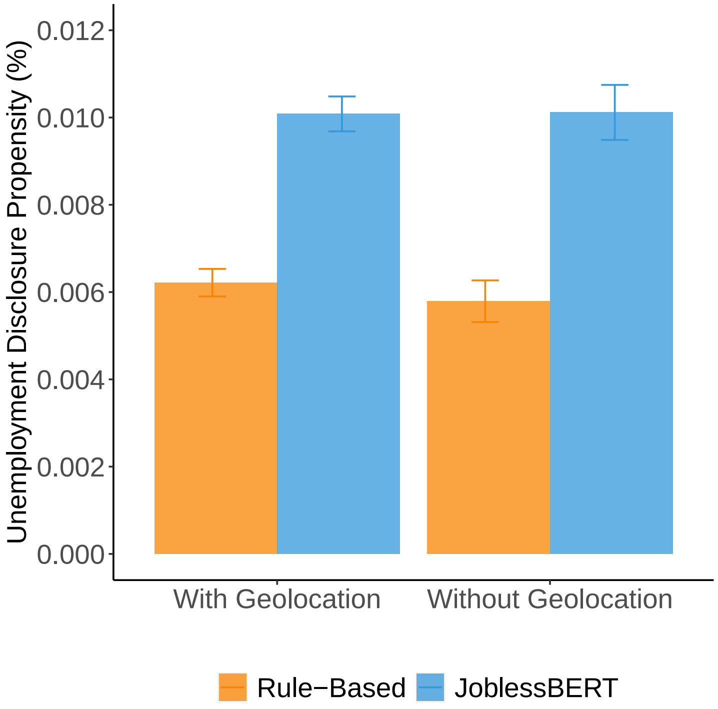

Supplementary Fig. S6: Propensity of unemployment disclosure by users with and without geolocation.

Average disclosure propensity by users with and without information on their geolocation. Propensities measured as the share of a user's tweets classified as unemployment-related. Overlaid lines to each bar represent 95% confidence intervals.

S7. Real-time forecast comparison during the March 2020 COVID-19 shock.

Supplementary Fig. S7 summarizes a real-time comparison of UI claims forecasts during the unprecedented spike in unemployment caused by the onset of the COVID-19 pandemic. We focus on the week ending March 21, 2020, when initial UI claims surged to 2.9 million. Panel A reports predictions made two days before the end of the measurement week (Friday, March 20), comparing the professional consensus forecast with estimates derived from the rule-based and JoblessBERT models using Twitter disclosures. Panel B presents the updated JoblessBERT forecast made one day before the official data release. The results illustrate the model's responsiveness to emerging labor market shocks: both Twitter-based models, and especially JoblessBERT, captured the magnitude of the spike far more accurately than the consensus forecast, despite relying solely on social media signals. This example highlights the potential of JoblessBERT to serve as an early warning system under extreme conditions.

Model	Predicted Claims	Forecast Error	Percent Error
Panel A: Forecasts made 2 days before week end (March 20, 2020)			
Consensus Forecast	0.33 million	-2.57 million	-88.8%
Rule-Based Model	2.32 million	-0.58 million	-20.5%
JoblessBERT Model	2.66 million	-0.24 million	-8.3%
Panel B: Forecasts made 1 day before official release (March 25, 2020)			
Consensus Forecast	0.33 million	-2.57 million	-88.8%
Rule-Based Model	2.52 million	-0.38 million	-13.1%
JoblessBERT Model	2.80 million	-0.10 million	-3.4%
Actual UI Claims (Reported)	2.90 million		

Supplementary Fig. S7: Predictions of UI claims for the week ending March 21, 2020. This table compares model predictions of U.S. unemployment insurance (UI) claims for the week ending March 21, 2020, following the COVID-19 pandemic declaration. **(A)** reports forecasts made on Friday, March 20, two days before the end of the measurement week. **(B)** shows JoblessBERT's updated forecast made on Wednesday, March 25, the day before the official UI claims release. Forecast error is defined as predicted claims minus actual claims (2.90 million). Percent error is the forecast error divided by actual claims.

S8. Model Performance by Subsample.

To evaluate whether the predictive strength of the Twitter-based unemployment signal depends on the level of media coverage, we partition the sample into quartiles based on a composite Google Trends index reflecting public interest in unemployment-related terms. We then compute out-of-sample mean squared errors (MSE) for each forecasting model across these quartiles.

The composite score used to define media attention quartiles is based on weekly Google Trends data for the search term "unemployment" in the United States. The index reflects relative search interest over time, where a value of 100 represents peak popularity during the sample period, 50 indicates half the peak search volume, and 0 means insufficient data. This normalized index is used to partition the testing period into quartiles of media attention, with quartile 1 representing the lowest and quartile 4 the highest levels of public interest.

Supplementary Fig. S8A shows that while forecast accuracy is highest during periods of elevated media attention (top quartile), the Twitter-based models retain substantial predictive power even during low-attention periods. For example, the JoblessBERT model exhibits only a modest decline in performance from the highest to lowest quartiles, suggesting the signal remains informative even when unemployment is not widely discussed in the news.

We also examine model performance across calendar years in the testing period (2020–2022), shown in Supplementary Fig. S8B. Forecast accuracy was highest in 2020, coinciding with the onset of the COVID-19 pandemic and a surge in unemployment-related discourse. In 2021 and 2022, forecasting errors increased somewhat across all models, consistent with reduced labor market volatility and lower public attention to unemployment. Nonetheless, the Twitter-based models, particularly JoblessBERT, consistently outperform the rule-based model and benchmark forecasts in all years, demonstrating robustness of the approach even as macroeconomic conditions and platform dynamics evolve.

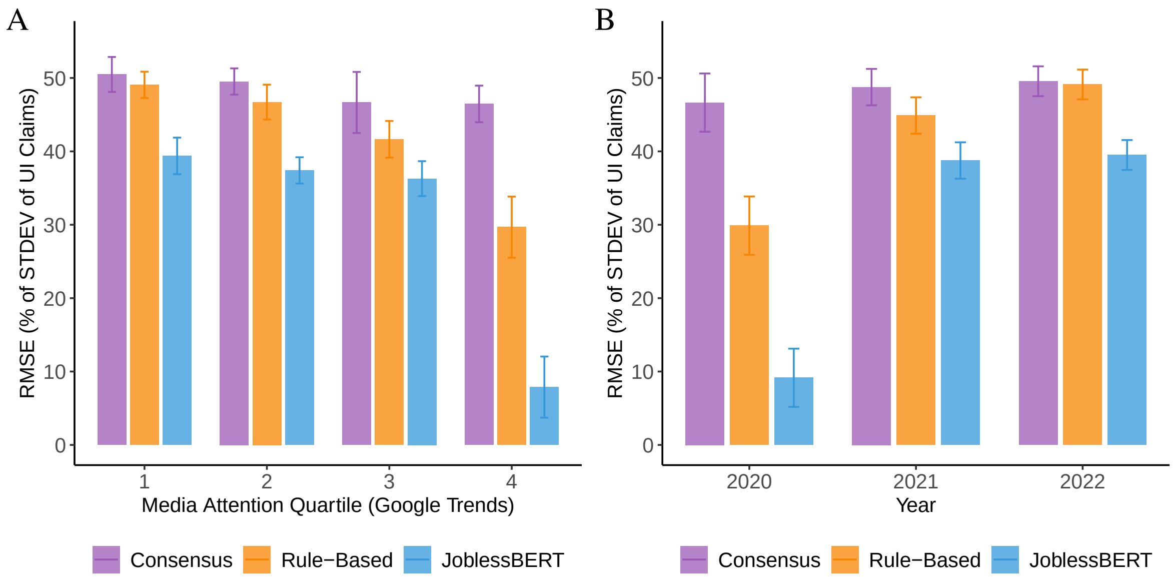

Supplementary Fig. S8: Model performance by subsample.

(A) Mean squared error (MSE) for unemployment forecasts with horizons as of the end of the measurement week, where the testing sample is partitioned into quartiles based on Google Trends composite scores for unemployment-related search activity. Q1 represents periods of lowest media attention, Q4 represents periods of highest media attention. **(B)** MSE for unemployment forecasts by calendar year (2020–2022). Error bars reflect 95% confidence intervals. Across both panels, lower MSE indicates higher forecast accuracy.

S9. Comparison with Enhanced National-Level Baseline Model with Google Trends.

In this section, we construct an alternative forecasting baseline ("GoogleTrends") that incorporates a national Google Trends index capturing media attention to unemployment-related keywords. This enhanced baseline reflects what a reasonably informed forecaster might observe in real time without relying on social media signals. Supplementary Fig. S9 compares the forecasting accuracy of JoblessBERT and the rule-based model to this new baseline. While the stronger baseline improves upon the consensus model, JoblessBERT still provides substantial gains in predictive accuracy in most states.

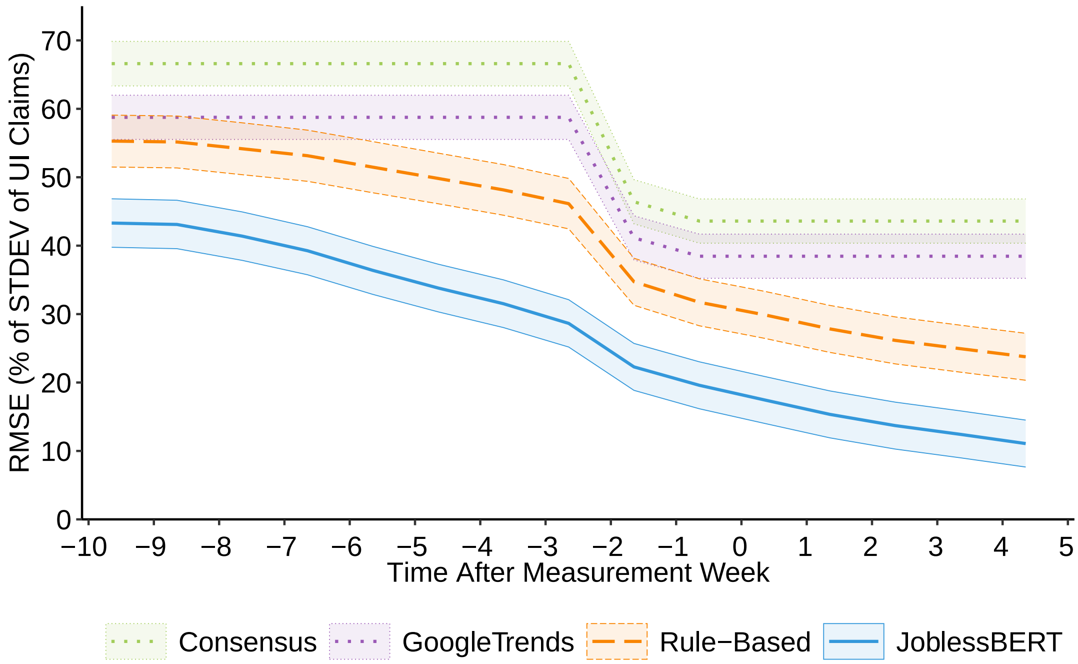

Supplementary Fig. S9: Enhanced national-level baseline model with Google Trends. National-level RMSE comparison against an enhanced baseline ("GoogleTrends") incorporating historical UI claims, national consensus forecasts, and Google Trends unemployment indices. The figure compares four models: consensus, GoogleTrends, rule-based, and JoblessBERT models. The horizontal axis represents the number of days relative to the end of the measurement week (day 0), and vertical axis reflects forecast accuracy. Forecasting accuracy is measured in root mean squared error (RMSE) as a share of the standard deviation of UI claims. Shaded bands around point estimates denote 95% confidence intervals.

S10. Comparison with Enhanced State-Level Baseline Model with Google Trends.

In this section, we construct an alternative forecasting baseline ("GoogleTrends") that incorporates a state-specific Google Trends index capturing media attention to unemployment-related keywords. Supplementary Fig. S10 compares the forecasting accuracy of JoblessBERT and the rule-based model to this new baseline across U.S. states. While the stronger baseline improves upon the autoregressive model, JoblessBERT still provides substantial gains in predictive accuracy at the state level.

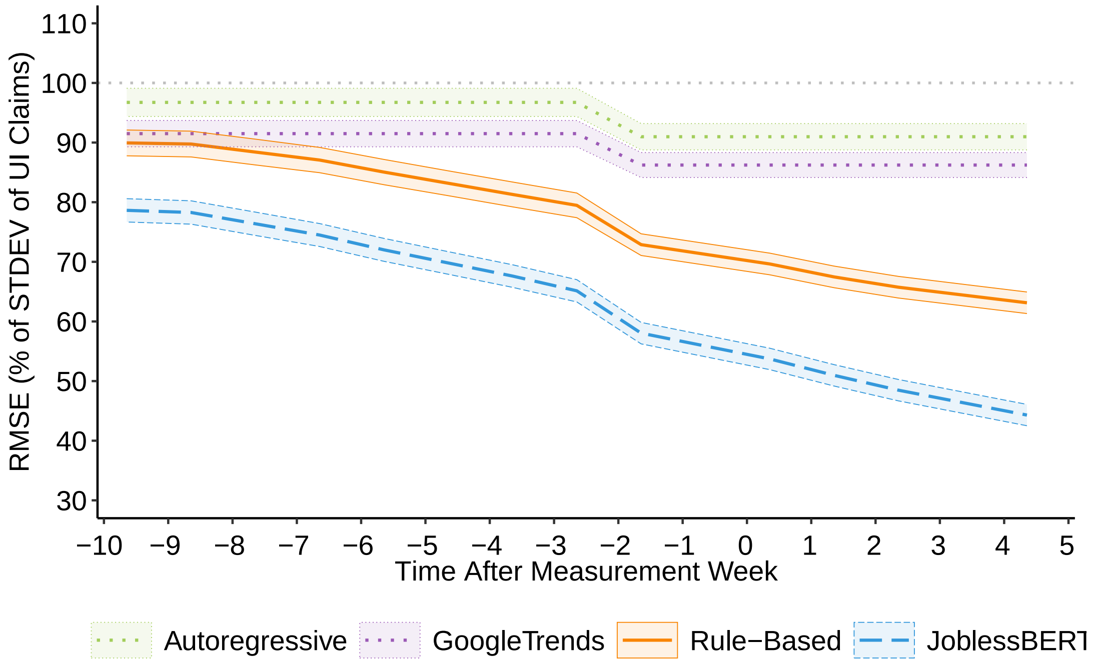

Supplementary Fig. S10: Enhanced state-level baseline model with Google Trends. State-level RMSE comparison between JoblessBERT and enhanced baseline ("GoogleTrends") incorporating historical UI claims and Google Trends unemployment indices. The figure compares four models: autoregressive, GoogleTrends, rule-based, and JoblessBERT models. The horizontal axis represents the number of days relative to the end of the measurement week (day 0), and vertical axis reflects forecast accuracy. Forecasting accuracy is measured in root mean squared error (RMSE) as a share of the standard deviation of UI claims. Shaded bands around point estimates denote 95% confidence intervals.

S11. City-level Predictions of Unemployment Insurance Claims in the U.S.

To push our approach to the limit, we evaluate the performance of our models at the city level using data from 45 cities across the U.S. (See Materials and Methods). As shown in Fig. S11A, JoblessBERT's model continues to outperform all other models at the city level, with RMSE reductions of 23.0% over the baseline and 8.4% over the rule-based model, respectively ($P < 0.001$). Comparing the best-performing models at the city and state levels (i.e. JoblessBERT the day prior to data release), we find that state-level predictions are significantly more accurate than city-level predictions, leading to RMSEs of 0.45 and 0.73 standard deviations, respectively ($P < 0.001$). This suggests that our approach begins to reach a performance limit at the city level. Supplementary Fig. S11B evaluates the RMSE of city-level predictions (holdout cities) as a function of the forecasting horizon. To further investigate model behavior at the limit, we examine predictive performance as a function of variability in claims and the adoption of Twitter at the city level. Panels C and D of Fig. S11 show city-level RMSE as a function of the average change in UI claims (panel C), and as a function of the estimated Twitter penetration rate (panel D). Clearly, performance varies across cities, but an overall trend is observed in panels C and D: predictions are more accurate when UI claims are changing and when Twitter's adoption is higher.

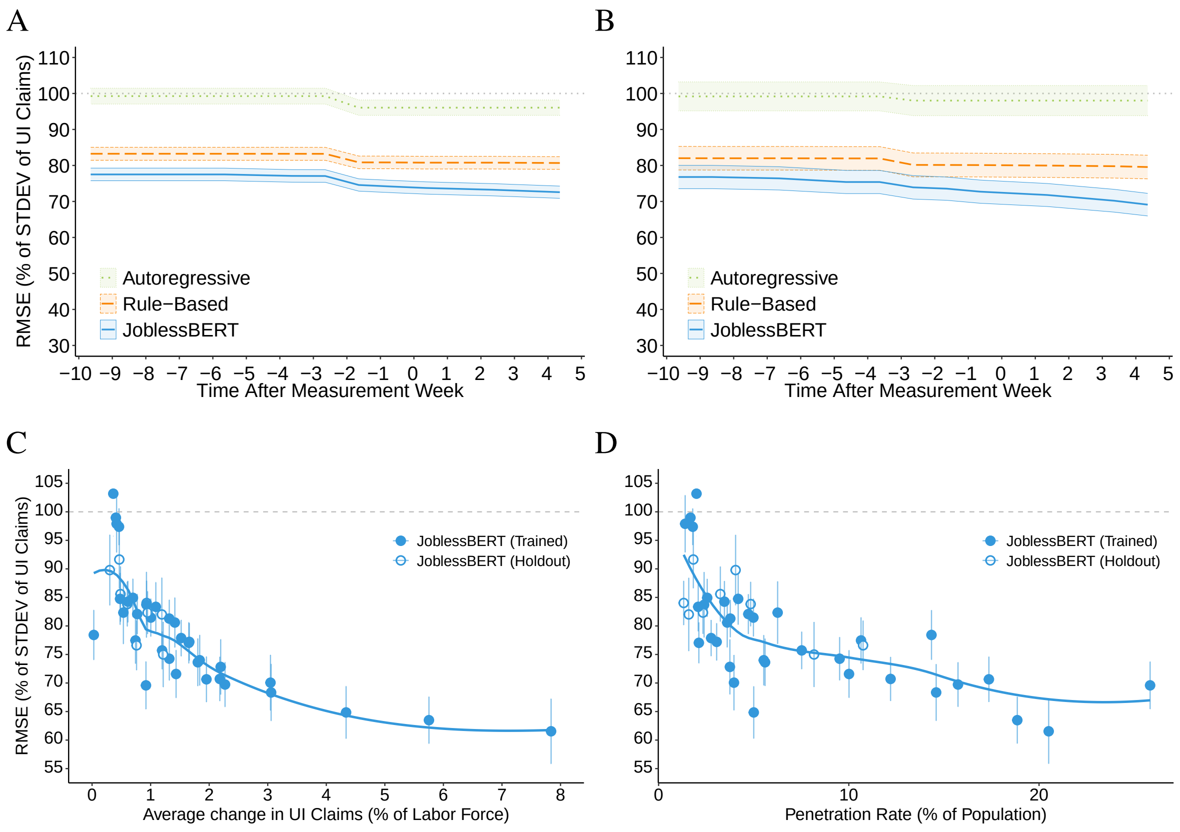

Supplementary Fig. S11: City-level predictions of U.S. unemployment insurance claims. **(A)** RMSE of city-level predictions (trained cities) as a function of the forecasting horizon. **(B)** Forecast accuracy for holdout cities not used in model training, shown as a function of the number of days relative to the end of the measurement week (day 0). **(A-B)** compares three models: an autoregressive baseline, the rule-based model, and JoblessBERT. RMSE is again normalized by the standard deviation of actual UI claims. Shaded bands around point estimates denote 95% confidence intervals. **(C)** RMSE by city as a function of the average change in UI claims, averaged over a two-week period prior to release. **(D)** RMSE by city as a function of the Twitter penetration rate, averaged over a two-week period prior to release. For all panels, RMSE is normalized by the standard deviation of actual UI claims in the respective city. Shaded bands and vertical lines around point estimates denote 95% confidence intervals.
